# A GRU–CNN model for auditory attention detection using microstate and recurrence quantification analysis

**DOI:** 10.1038/s41598-024-58886-y

**Published:** 2024-04-17

**Authors:** MohammadReza EskandariNasab, Zahra Raeisi, Reza Ahmadi Lashaki, Hamidreza Najafi

**Affiliations:** 1 College of Science, Utah State University, Logan, USA; 2 Department of Computer Science, University of Fairleigh Dickinson, Vancouver Campus, Vancouver, Canada; 3 Department of Computer Engineering, Faculty of Electrical and Computer Engineering, University of Tabriz, Tabriz, Iran; 4 Biomedical Engineering Department, School of Electrical Engineering, Iran University of Science and Technology, Tehran, Iran

**Keywords:** Auditory attention detection, GRU–CNN, EEG, Microstate analysis, Machine learning algorithms, Multivariate features, Recurrence quantification analysis, Auditory system, Cognitive neuroscience, Biomedical engineering

## Abstract

Attention as a cognition ability plays a crucial role in perception which helps humans to concentrate on specific objects of the environment while discarding others. In this paper, auditory attention detection (AAD) is investigated using different dynamic features extracted from multichannel electroencephalography (EEG) signals when listeners attend to a target speaker in the presence of a competing talker. To this aim, microstate and recurrence quantification analysis are utilized to extract different types of features that reflect changes in the brain state during cognitive tasks. Then, an optimized feature set is determined by employing the processes of significant feature selection based on classification performance. The classifier model is developed by hybrid sequential learning that employs Gated Recurrent Units (GRU) and Convolutional Neural Network (CNN) into a unified framework for accurate attention detection. The proposed AAD method shows that the selected feature set achieves the most discriminative features for the classification process. Also, it yields the best performance as compared with state-of-the-art AAD approaches from the literature in terms of various measures. The current study is the first to validate the use of microstate and recurrence quantification parameters to differentiate auditory attention using reinforcement learning without access to stimuli.

## Introduction

Humans are able to concentrate on a special speaker in a cocktail party environment. This phenomenon describes the auditory capability of the brain during the attention to a target among others which is known as auditory selective attention. Auditory attention plays a salient role in vision and auditory perception and also assists us in concentrating on a single speaker. It is yet unclear what happens in the brain to facilitate the attentional process and separate a specific voice or sound from the background. The research on auditory selective attention was introduced first by Cherry^1^. After that, other researchers presented several dichotic^2^ and binaural^3^ methods to examine the mechanism of auditory attention detection (AAD) in adverse real conditions^4^. There are numerous applications regarding AAD modeling such as brain-computer interface (BCI) systems^5^, robotics, controlling sound recording devices^6^, and neuro-steered hearing aids^7^. As a golden aim, the notion of AAD can also be employed in a neuro-steered hearing prosthesis, where the device can separate and amplify the attended speech of a hearing-impaired listener placed in a cocktail party scenario.

Auditory attention has been revealed as a neural unit that is involved with high-level cognitive processing in the cerebral cortex^8^. This process can be decoded from the recordings of brain activities such as electroencephalography (EEG)^9^, magnetoencephalography (MEG)^10^, and functional magnetic resonance imaging (fMRI)^11^. EEG is a more popular tool in cognitive neuroscience studies because of its applicability and accessibility in real-time measurements. Recently, various methods have been developed to find selective auditory attention using EEG signals. Extracting informative features^12^, backward mapping^13–16^, and forward mapping^17–20^ are three main techniques that are utilized to detect the attended speech/speaker. Backward and forward mapping methods detect the attended and unattended speech envelopes via designing speaker-specific decoders in the low-frequency range (1–8 Hz) where EEG corresponds to the spectrum of speech envelopes in this frequency range^17–19,21^. It is clear that for AAD, forward and backward mapping require clean speech to compute the correlation between stimuli envelopes and EEG data; a condition that never happens in realistic acoustic environments. It has been also indicated that the accuracy of decoders in backward mapping procedures depends on the trial length or temporal resolution of stimuli (e.g., shorter trial lengths such as 10 s are preferred over the most reported 60 s trial length^22^). In contrast to these approaches, the informative features technique does not require clean auditory stimuli and this characteristic makes it applicable in real-life conditions suchlike a cocktail party. Many features derived from EEG were exploited for auditory attention classification^23–27^. Although many researchers have introduced various features for attention detection, such features could not resolve inconsistencies or ambiguities in EEG interpretations. Furthermore, such features fail to utilize EEG in millisecond temporal resolution. So, it is essential to develop diagnostic features that can exploit it.

On the other hand, several studies have attempted to use EEG microstate analysis to develop diagnostic features that can exploit temporal resolution in milliseconds. For example, Lehmann et al.^28–30^ demonstrate that the existence of a quasi-stable microstate by segmenting spontaneous EEG at the sub-second level produces stable and evenly patterned results at 80–120 ms intervals. Many previous researchers have revealed microstates change in various diseases and mental states such as anxiety disorder^31^, neurodegenerative disorder^32–34^, sleep^35^, mood disorder^36^, schizophrenia^37^, and emotion revelation^38^, physical exercise^39^, insomnia^40^, hearing loss^41^. Since the EEG microstates provide different states of brain activity with high interpretability (i.e., A, B, C, and D are known to be associated with auditory, visual, default mode, and dorsal attention), it seems to be advantageous to use this feature in exploring the selective auditory attention detection. As regards, multivariate analysis can improve reliability and validity in multiple dependent and independent variables, therefore, it can utilize all microstate-feature information and determine new patterns to enhance the classification model. By applying the recurrence quantification analysis (RQA) approach to detect dynamic changes in microstates, we can create a more powerful model to reveal the listener’s auditory attention. Machine learning algorithms perform multivariant analysis to distinguish between EEG recordings of subjects attending to a target speaker and those attending to another speaker and present a practical application. In summary, reinforcement learning-based microstate and recurrence quantification analysis is a novel approach to auditory attention detection.

The main problem of auditory attention detection in previous studies is that these methods do not have high performance online from only EEG signals without access to clean speech. The present work introduces a new dynamic method based on microstate and recurrence quantification analysis to detect auditory attention in each second and millisecond of EEG signals.

In this paper, first, we hypothesize that microstate features (MS) based on their archetypes are useful for AAD. Then, microstate features are obtained from the EEGs of the subjects during the auditory attentional task. Next, RQA is applied to investigate the dynamic changes of selected microstate features. Multivariant analysis by extracting dynamic characteristics from optimal features is performed to preserve the important information. Classification performance is obtained to differentiate between attended and unattended tasks using the extracted features. Finally, the best results of multi-level features are reported and compared with other researches.

The organization of the paper is as follows. In Section "Literature Review", a comprehensive literature review is presented. Section "Material and Methods" explains the material and the methodologies, including the description of EEG data and the proposed AAD model. Also, the details of feature extraction approaches and classifiers are presented in this section. Section "Experiments and evaluations" presents the experimental setup and evaluation criteria. Section "Results and Discussion" discusses the findings of the experiments and compares the proposed method with recent baseline AAD algorithms from the literature. Finally, the concluding findings of the research work are given in Section "Conclusion".

## Literature review

Various experiments have verified the feasibility of decoding selective auditory attention in multi-talker environments using EEG signals and speech stimuli^12,27,42–50^. This section comprehensively reviews the related works, showcasing the pivotal role of features and deep learning in auditory attention detection.

In 2012, Mesgarani and Chang^21^ showed that it is possible to decode auditory attention in multi-talker scenarios from brain signals. Here, speech spectrograms reconstructed based on cortical responses to the mixture of speakers reveal the salient temporal and spectral features of the attended speaker, as if subjects were listening to that speaker alone. Therefore, both attended words and speakers can be decoded by a simple classifier trained on an example of single speakers. O’Sullivan et al^46^. showed that single-trial unaveraged EEG data can be decoded to determine attentional selection in a naturalistic multi-speaker environment. They found that there is a significant correlation between the EEG measure of attention and performance on a high-level attention task. Also, neural processing at ~ 200 ms as being critical was identified for solving the cortical party problem to decode attention at individual latencies. Previous approaches in decoding the auditory attention have mainly focused on linear mappings between the sound stream cues and EEG responses^51,52^. More specifically, the mapping from auditory stimuli to cortical responses is typically referred to as the forward model or multivariate temporal response function (mTRF). It can be used to map in both the forward and backward direction to perform response function estimation and stimulus reconstruction, respectively. Fuglsang et al^53^. used the mapping from cortical responses to acoustic features as the backward model or stimulus reconstruction to decode the attentional selection of listeners in multi-talker scenarios. With reverberant speech, they observed a late cortical response to the attended speech stream that encoded temporal modulation in the speech signal without its reverberation distortion. de Cheveigne et al^54,55^. proposed an alternative to both forward and backward mapping, namely canonical correlation analysis (CCA). The performance of these linear decoding approaches decreases significantly when operated at low latency settings. Cai et al^56^. introduced an EEG-graph net that exploits the topology of the human brain to perform auditory spatial attention detection from EEGs. Their results showed that EEG-graph net significantly outperforms in terms of decoding performance.

These studies have two deficiencies: (1) the accuracy of AAD models is fairly low, approximately 60%, over a data window with a length of 1 s, and its mapping and correlation evaluation process are not jointly optimized for attention detection. This issue motivated the researchers to propose non-linear models to detect the attended speakers based on EEG signals to realize low-latency AAD. Hence, Deckers et al^48^., Ciccarelli et al^57^., and Vandecappelle et al^58^. presented AAD models based on a convolutional neural network (CNN) to detect attended speakers. However, these non-linear AAD approaches disregarded valuable temporal information of EEG signals and more advanced decoding strategies are needed to realize robust real-time AAD.

## Material and methods

### Participants and EEG recording

In this study, all experiments are conducted on two publicly available databases, namely DTU^59^ and KUL databases^60^.

1. *DTU database*: This dataset was published in^59^ and acquired from 44 subjects (age 51–76) where 22 of them were hearing-impaired (19 right-handed, 9 females) and the rest were normal-hearing (16 females, 18 right-handed) listeners. All the EEG signals were collected with a sampling frequency of 512 Hz from the subjects in two ways: ear-EEG with 6 electrodes (three for each ear) and 64-channel EEG scalp recorded by the BioSemi Active-Two system. During the EEG recording, the subjects listened to one of the two simultaneous speech streams or a single speech stream in the quiet condition. Two different audiobooks in Danish read by a female and a male speaker (denoted as ‘Spk1’ and ‘Spk2’ in further analysis) were taken as speech stimuli presented in 48 trials each with $\sim$ 50 s length and 65 dB SPL. The audio files were filtered by a low-pass second-order Butterworth filter to avoid excessive high-frequency amplification for subjects with low audiometric thresholds. Each subject listened to either a single talker or two competing talkers during the 48 trials (all recording time for a listener is 50 s × 48). Sixteen trials were presented with a single talker (8 trials read by a female and 8 trials read by a male) and 32 trials were played with a multi-talker (one male and one female). In the multi-talker trials, the two speech streams were presented at the same loudness level to allow unbiased attention decoding. The audio files were presented at $\pm 90^{ \circ }$ azimuthal positions by non-individualized head-related transfer functions (HRTFs) and preprocessed. Listeners were prompted to answer 4 questions with multiple-choice comprehension regarding the content of the attended speech stream (for details see^59^). It should be noted that one subject (number 24) was excluded from our analysis due to signal interruption during one of the trials.
2. *KUL database*: This dataset was published in^60^ and consists of 16 normal-hearing subjects (age 17–30) where 8 of them were male and the rest were female. The speech stimuli include four Dutch stories, narrated by three male and female speakers. The audio files were presented dicotically at $\pm 90^{ \circ }$ azimuthal positions by HRTF filtering. 64-channel EEG signals were recorded using a BioSemi ActiveTwo device at a sampling rate of 8192 Hz. A total of 72 min of EEG was recorded per subject, approximately 36 min per attended ear. All stimuli were normalized to have the same root-mean-square value and the attended stories were randomized across subjects. It is noted that the audio signals were filtered by a low pass filter at 4 kHz.

### Proposed auditory attention detection method

**Figure 1:**
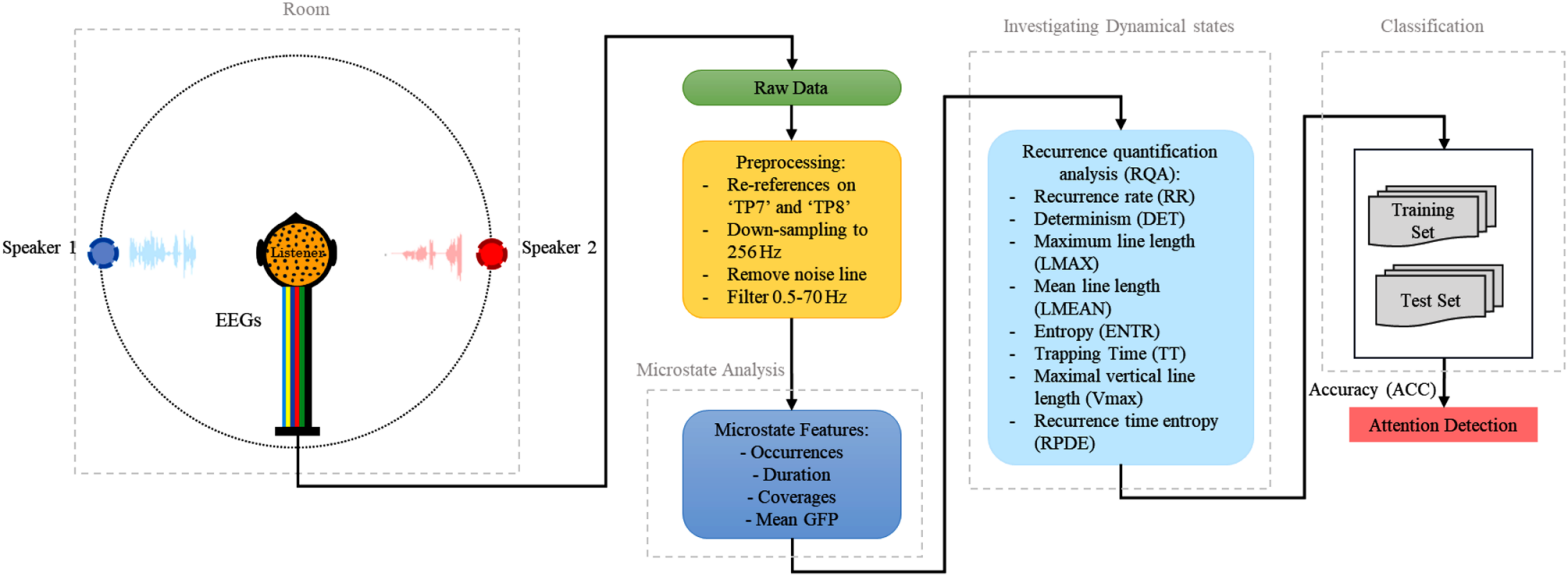
The proposed AAD method based on microstate and recurrence quantification analysis.

In the present work, auditory attention activity is assayed during the concentration on a narrator in multi-talker scenarios. Figure 1 depicts the proposed AAD procedure in this research. Multivariate dynamic features based on EEG microstates are extracted in multi-step and classified from the EEG signals to model the AAD process in the presence of a competitive talker. Machine learning algorithms, which learn from input to make data-driven decisions, are now widely used for the analysis of EEGs. These learning algorithms are employed to train a model and develop related models with the input high-level feature vectors and lend themselves to prediction. We exploit the K-nearest neighbor (KNN), support vector machine (SVM), long short-term memory (LSTM), bi-directional long short-term memory (Bi-LSTM), and Q-Learning to construct the AAD model.

#### EEG preprocessing

The sampling frequency of both EEG datasets is resampled to 256 Hz. EEG data is carefully checked for body movements, eyes blinking, muscle activity, and technical artifacts. A 0.5–70 Hz band-pass finite impulse response (FIR) Butterworth filter is used to eliminate the interference of high-frequency noises with the 2112th and 212th order, respectively. The digitized EEG signals are re-referenced to the average of electrodes TP7 and TP8.

#### EEG microstate analysis

EEG microstate analysis is a strong tool to study the temporal and spatial dynamics of human brain activity^61^. Microstate analysis reflects cortical activation for quasi-stable states in 60–120 ms which is important for investigating brain dynamics^62^. The pre-processed EEG data is analyzed by MNE^63^ to detect EEG microstates and compute the characterization. A standard procedure for EEG microstate analysis includes four stages: (1) candidate topography extraction, (2) EEG microstate detection, (3) EEG microstate segmentation, and (4) microstate feature extraction. In the first step, the Global field power (GFP) is used to characterize the global pattern of neuro-electrical and dynamic fluctuations of the brain which is defined as:

1 $$GFP(t) = \sqrt {(\sum\nolimits_{i}^{N} {(x_{i} (t) - \overline{x}(t)^{2} )/N} )} ,$$

where $x_{i} (t)$ and $\overline{x}(t)$ are the instantaneous and mean potentials across *N* electrodes at time *t*.

In the second stage, topographies of each electric activity at the local GFP maximum point are recognized as a discrete EEG state and signal evolution is a series of such states. The successive microstates are derived from the EEG analyzed based on local maximum points of GFP in discrete states. In the next stage, using clustering methods, all microstates can be determined according to microstates patterns. The patterns have enabled many studies that uncovered their function and applied them to various disorders^35,37,64–66^. Most studies in this field have reported 4 patterns of microstate topographies to represent brain activity measured using recording EEG. These four topographies included type A (right-frontal left posterior), type B (left-frontal right-posterior), type C (midline frontal-occipital), and type D (midline frontal), respectively^37^. Single topography remained quasi-stable for durations of about 80–120 ms before dynamically transitioning to another topography. Finally, when an EEG is considered to be a series of topographies of electric potentials that evolve, the entire recording can be studied using a set of topographies that dynamically fluctuate amongst themselves at discrete time points.

**Figure 2:**
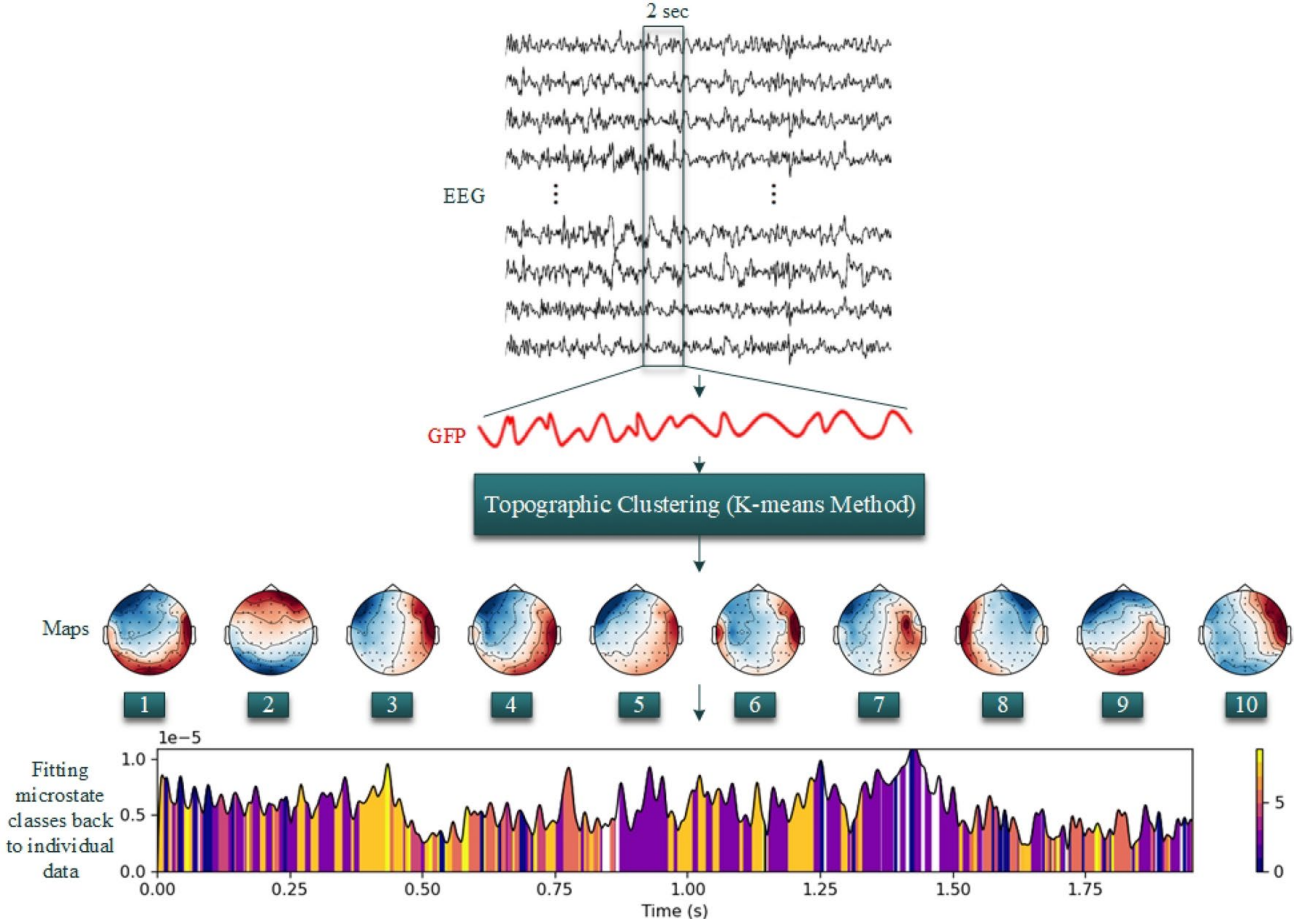
Schematic flowchart of the EEG microstate analysis using MNE. The GFP of each sampling point is calculated and all topographic maps at the local GFP maxima are obtained. The K-means clustering analysis method is used to analyze topographic maps to obtain optimal microstate classes. At the bottom, microstate temporal sequences are obtained by fitting microstate classes back to complete EEG data.

Figure 2 displays the microstate analysis for 2 s of attention task on EEG signal. At first, the GFP (depicted as a red line) is calculated at each given time duration as the spatial standard deviation (std). In the second step, the K-means clustering approach is executed on the scalp topographies of each input data. Several studies use the K-means clustering by the cross-validation (CV) metric to demonstrate that the optimal numeral of classes within subjects was four^35,67^. We set the numeral of clusters from 2 to 10 and the optimum set of classes is selected according to the maximum values of global explained variance (GEV). In the subsequent step, momentary maps of each group (group one: attending to ‘Speaker 1’ and group two: attending to ‘Speaker 2’) are separately categorized into 10 microstate clusters. Eventually, the generated class-labeled group maps are used as schema to allocate original individual successive EEG series of each listener to 10 microstate patterns shown in this figure.

**Figure 3:**
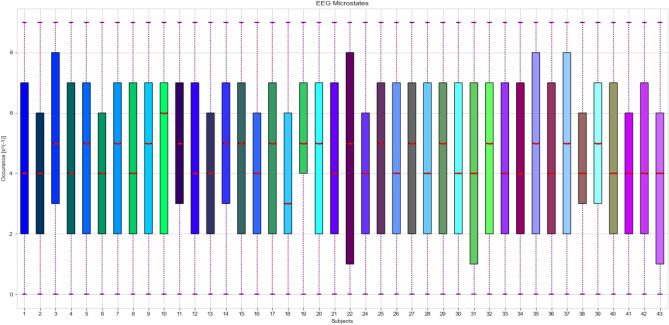
Changes in occurrence values of microstate features depend on each subject of DTU database and performance. Boxplots represent the first quantile, third quantile, and median values.

In every microstate, four types of specification, namely, mean GPF, occurrence, duration, and coverage are computed. Mean GFP is determined as the middle GFP for a state. The occurrence is interpreted as the middle frequency of the detected states. Duration is explained as the middle length of states per unit. Coverage describes the percentage of each state appearing in every epoch. Figure 3 illustrates the occurrence values of EEG states which vary between subjects.

Since the clustering algorithm diminishes the complete set of spatial patterns, four methods derived from classical clustering algorithms, namely, independent component analysis (ICA)^68^, principal component analysis (PCA)^68^, atomize and agglomerate hierarchical clustering (AAHC)^69^, and k-means^70^ algorithms. The GEV values for different numbers of EEG microstates are given in Table 1. Here, the optimal numeral of microstates is determined and then, their labels are sorted into a sequence by using four clustering algorithms and GEV criteria. GEV measures how similar each EEG sample is to the microstate prototype and it has been assigned to where the higher GEV is better. In the microstate analysis, the maximum value of GEV was selected after 10 iterations of re-run.

**Table 1:** GEV values using ICA, AAHC, PCA, and K-means clustering for different numbers of *N*.

Number of Microstates (N)		ICA	AAHC	PCA	K-Means (max iteration = 500)	K-means (max iteration = 1000)
DTU	2	0.621	0.528	0.635	0.706	0.477
	3	0.348	0.612	0.639	0.773	0.593
	4	0.261	0.643	0.641	0.794	0.617
	5	0.353	0.652	0.642	0.806	0.655
	6	0.249	0.681	0.643	0.816	0.685
	7	0.366	0.694	0.643	0.820	0.704
	8	0.332	0.702	0.643	0.826	0.712
	9	0.231	0.709	0.643	0.832	0.718
	10	0.315	0.717	0.643	**0.834**	0.729
KUL	2	0.473	0.254	0.306	0.127	0.399
	3	0.488	0.255	0.409	0.278	0.538
	4	0.490	0.258	0.551	0.346	0.654
	5	0.501	0.276	0.655	0.332	0.752
	6	0.547	0.316	0.665	0.415	0.774
	7	0.585	0.440	0.795	0.505	0.779
	8	0.616	0.5381	0.714	0.613	0.794
	9	0.646	0.638	0.823	0.758	0.800
	10	0.653	0.745	0.847	**0.865**	0.801

Significant values are in [bold].

#### Recurrence quantification analysis (RQA)

**Figure 4:**
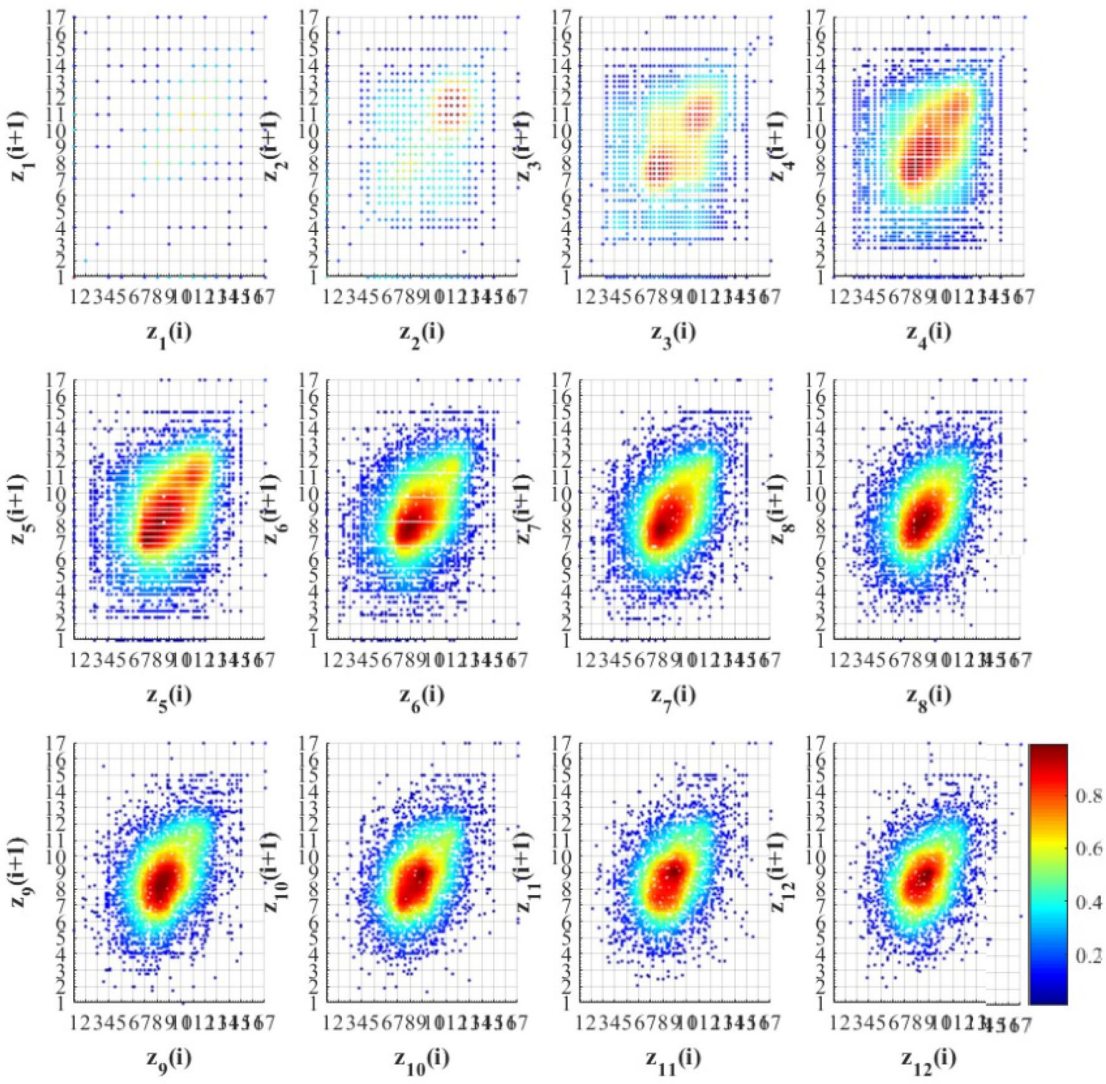
Example of recurrence plots (RP) with 12 different scales for the GFP extracted from EEG of DTU database.

To derive some useful non-linear dynamic attributes from the various states of the EEG signal, an RQA is performed^71^. Several studies have used RQA parameters to analyze EEG signals and quantify the cortical function at sleep apnea syndrome^72^, different sleep stages^73^, epileptic identification^74^, and tactile roughness discrimination^75^. It has the capability to extract non-linear characteristics of signals and quantify the complex and deterministic behavior of EEG signals. Recurrence refers to the trajectory returning to the former state in the phase space, which is generally constructed from a time-series signal using a time-embedding method. A recurrent plot (RP) was used to visualize the amount of recurrence in a multi-dimensional dynamic system by simply illustrating a dot square matrix in a two-dimensional space (see Fig. 4). In Eq. (2), $R$ is calculated for each sample, $i,j$ of the time series $x$, under the predefined threshold distance $\varepsilon$^76^:

2 $$R_{i,j} = \Theta \left( {\varepsilon - \left\| {x_{i} - x_{j} } \right\|} \right),\;\;\;i,j = 1,\,2, \ldots \,,\,N,$$

where $\Theta \left( . \right)$ is the Heaviside function, $\left\| . \right\|$ is the maximum norm, and *N* is the number of samples in the phase space trajectory. The distance in the phase space between $x_{i}$ and $x_{j}$ falls within the $\varepsilon$, two samples are considered to be recurrences, indicated as $R_{i,j}$. Several features can be obtained to quantify the RP where each of them indicates a specific characteristic of the signal. In this work, the following features are extracted from the RP:

*Recurrence Rate (RR)*: This index measures the percentage of recurrence points in the RP which is calculated as^77^:

3 $$RR = \frac{1}{{N^{2} }}\sum\nolimits_{i,j = 1}^{N} {R_{i,j} .}$$

*Determinism (DET)*: This measure shows the percentage of recurrence points in the diagonal lines in the RP^78^. Higher values of this index indicate that the signal $x$ has a deterministic nature with higher probability. It is computed by:

4 $$DET = \frac{{\sum\nolimits_{{l = l_{\min } }}^{N} {l\,.\,P(l)} }}{{\sum\nolimits_{l = 1}^{N} {l\,.\,P(l)} }},$$

where $l$ and $l_{\min }$ are the length of the diagonal line and minimum value, respectively. $P(l)$ is the frequency distribution of the length $l$.

*Maximum line length (L*_MAX_*)*: The longest diagonal line on the RP is defined as *L*_MAX_:

5 $$L_{MAX} = \max \left( {\left\{ {l_{i} ;\,i = 1,\, \ldots ,\,N} \right\}} \right).$$

*Mean line length (L*_*MEAN*_*)*: The average length of the diagonal line on the RP is defined as L_MEAN_:

6 $$L_{MEAN} = \frac{{\sum\nolimits_{{l = l_{\min } }}^{N} {l\,.\,P(l)} }}{{\sum\nolimits_{{l = l_{\min } }}^{N} {P(l)} }}.$$

*Entropy (ENTR)*: This index measures the entropy of the diagonal line lengths and is calculated using Eq. 6. It discloses the RP complexity of the system structure^79^.

7 $$ENTR = - \sum\nolimits_{{l = l_{\min } }}^{N} {P(l)} \,.\,\ln P(l).$$

*Trapping Time (TT)*: The TT represents the length of time that the dynamics remain trapped in a certain state. TT is the average length of vertical lines in the RP, as below:

8 $$TT = \frac{{\sum\nolimits_{{v = v_{\min } }}^{N} {v\,.\,P(v)} }}{{\sum\nolimits_{{v = v_{\min } }}^{N} {P(v)} }}.$$

where $P(v)$ is the distribution of the length of vertical lines.

*Maximal vertical line length (V*_max_*)*: This feature indicates the maximal length of vertical lines in the RP structure and is computed as:

9 $$V_{\max } = \max \,\left( {\left\{ {v_{i} ;\,i = 1,\, \ldots ,N} \right\}} \right).$$

*Recurrence time entropy (RPDE)*: This parameter has been successfully applied in biomedical testing. RPDE has advantages in detecting subtle changes in biological time series such as EEG and indicates the degree to which the time series repeat the same sequence. It is defined as:

10 $$RPDE = - \ln T_{\max }^{ - 1} \sum\nolimits_{t = 1}^{{T_{\max } }} {P(t)\,\ln \,P(t)} .$$

#### Classification

To assess whether microstate features and recurrence quantification analysis are appropriate for auditory attention detection, several machine-learning algorithms are employed to compare classification performances.

- *K-nearest neighbor (KNN)*: a non-parametric supervised learning algorithm that recognizes the class of testing samples according to the near class of K-nearest training samples^80^. In the train stage, KNN receives feature vectors and class labels of the training dataset samples, whereas, in the testing stage, an unlabeled sample is categorized by assigning the label that is closest among the K training samples. The implementational details of the utilized KNN are shown in Table 2.
- *Support vector machine (SVM)*: operates as a supervised learning algorithm in classification analysis. When this algorithm is utilized for training, it builds a model for a given set of binary-labeled features as training data by maximizing the distance of hyperplanes. SVM maps input data into multidimensional space by a function and then, categorized groups that have maximum margins, and the region boundary by the hyperplanes^81^. Test samples are mapped into that multidimensional space and forecasted to belong to a class based on which side of the margin they drop. There is a constraint to prevent data points from falling into the margin which is called box constraint. This algorithm is widely utilized for classification problems due to its ability to manage huge data. The values of box constraint and types of kernel function affect the results of classification (more details in Table 2).
- *Long short-term memory (LSTM)*: shows great efficiency in feature sequence extraction and data classification in many implementations^82^. A simple LSMT cell consists of an input gate, output gate, forget gate, and the candidate cell. Each gate has an activation function with two weighted inputs: (1) the previous hidden state of the LSTM cell which is weighted by a recurrent weight, and (2) the current input which is weighted by an input weight where forgotten. Input and output gates have a sigmoid activation function and the cell candidate gate has a tangent hyperbolic function with bias values b. Therefore, the LSTM cell has two outputs: (1) the memory cell state, and (2) the hidden state^83^.
- *Bi-directional long short-term memory network (Bi-LSTM)*: as an extension of the traditional long-short term memory (LSTM)^84^, is trained on the input sequence with two LSTMs set up in reverse order. The LSTM layer reduces the vanishing gradient problem and allows the use of deeper networks as compared with recurrent neural networks (RNNs)^85^. The advantage of Bi-LSTM to CNN is its dependency on the sequence of inputs by taking the forward and backward paths into account. Table 2 shows the architecture of the utilized Bi-LSTM.
- *GRU–CNN Q-Learning (GCQL)*: is one of the reinforcement learning (RL) methods which is a numerical and iterative algorithm^42^. Q-learning attempts to estimate a value function that is closely related to the policy or which policy can be derived. Therefore, most RL problems can be solved by the Markov decision process (MDP) as a discrete-time state transition. Here, the current state $S$ and action $A$ of a system is independent to all previous states and actions $P(S_{t + 1} |S_{t} ,\,A_{t} )$. $P(...)$ is the probability of making a transition to the next state, $S_{t + 1}$ when the model receives action $A_{t}$ and state $S_{t}$^86^.

**Table 2:** Implementation setup of KNN, SVM, LSTM, and Bi-LSTM classifiers.

Classifier	Parameters	Value
KNN	Distance weight	equal
	Distance metric	Euclidean
	Number of neighbors	1
	Standardize data	true
	Solver	SMO
	Cross-validation	10
	Classification runs	50
SVM	Box constraint level	1
	Kernel function	Gaussian
	Kernel scale mode	1.1
	Standardize data	true
	Solver	SMO
	Cross-validation	10
	Activation function	Tanh
LSTM	Number of LSTM layers	5
	Number of max-pooling layers	1
	Number of fully-connected layers	1
	Number of SoftMax layers	1
	Number of batch size	512
	Dropout	0.2
	Activation function	Sigmoid
	Optimizer	Adam
Bi-LSTM	Number of LSTM layers	3
	Number of max-pooling layers	1
	Number of fully-connected layers	1
	Number of SoftMax layers	1
	Number of batch size	512
	Dropout	0.2
	Activation function	Sigmoid
	Optimizer	Adam
Number of epochs: 100		
Train-Test split: 70%-30%		

**Figure 5:**
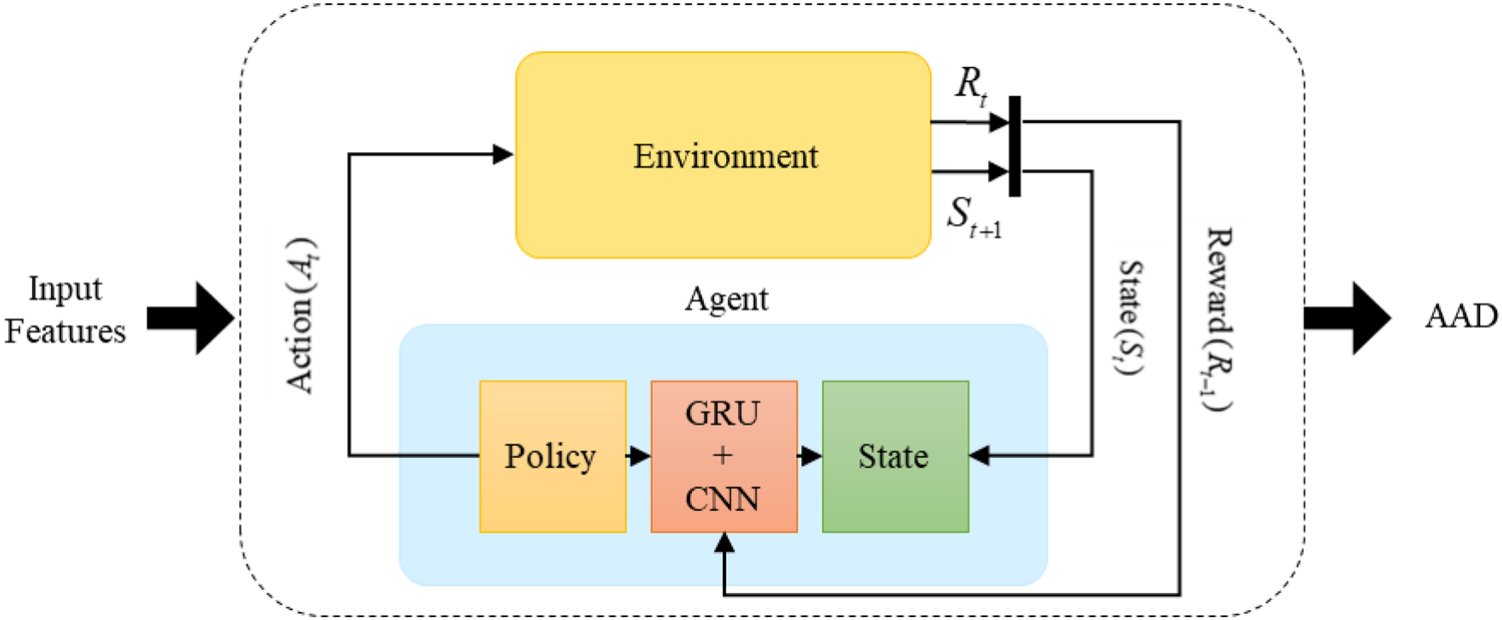
The block diagram of the proposed GCQL to improve estimating the value function.

The behavior of the model is described by a reward function $R_{t - 1}$, which measures the success or failure of an agent’s action in the environment. Here, GCQL represents an extension of QL to approximate optimal action-value function based on a gated recurrent network (GRU) and convolutional neural network (CNN) as the reinforcement learning method in the agent (see Fig. 5). As shown in this figure, the environment learns the optimal policy using the interactions between GRU and CNN in the agent. In other words, the RL algorithm employs this structure of neural networks as a function approximator.

It is noticed that CNN can learn representations and is very suitable for processing image data and RNN has memory ability in learning the non-linear features of sequence data such as EEG signals. GRU is a variant of RNN that can effectively alleviate the gradient disappearance and gradient explosion problem in the traditional RNN during training. It considers both historical information and new information when calculating the current state value. Therefore, the combination of GRU and CNN could improve the robustness of deep learning to decode small-scale EEG datasets and alleviate the overfitting phenomenon caused by insufficient data.

## Experiments and evaluations

### Experimental setup

Two experiments are conducted to assess the performance of the proposed AAD method based on microstate and RP features. The first experiment concerns the evaluation of the AAD procedure using different features extracted by microstate and recurrence quantification analysis, separately. In the second experiment, the efficiency of the MS and RQA features are assessed in different combinations.

To conduct the first experiment, the EEG data of 43 subjects during the 48 trials were selected to analyze the efficiency of the AAD. The four types of microstates and eight types of RP features are obtained on the input EEG signals on non-overlap windows along 256 samples. The extracted features are given to the classifiers (i.e., KNN, SVM, LSTM, Bi-LSTM, and GCQL) for detecting attended/unattended speech, separately. Here, seventy percent of data (i.e., 34 trials of all 48 trials recorded from each subject) is used as a training set and the rest is considered as the test set. In other words, both training and test data originated the same subject. In the second experiment, the combinations of MS and RP features as multivariate feature analysis are fed to the classifiers. This is performed to find appropriate features with high performance in attention detection from EEG signals.

To evaluate the performance of the proposed method, the recently developed attention detection system introduced by O’Sullivan et al^16^., Lu et al^25^., Ciccarelli et al^20^., Geirnaert et al^26^., Zakeri et al^27^., Cai et al^56^., and Niu et al^87^., are simulated and used as baseline systems from the literature.

### Evaluation criteria

The efficiency of the AAD algorithm is determined through three metrics: Accuracy, Sensitivity, and Specificity^88^. Accuracy (ACC) values show the overall detection correctness. Sensitivity (or true positive ratio: TPR) indicates the rate of correctly classified trials whereas Specificity (or true negative ratio: TNR) measures the rate of correctly rejected trials. Here, TP, TN, FP, and FN indicate true positive, true negative, false positive, and false negative predictions of the algorithm, respectively.

11 $$ACC = \frac{TP + TN}{{TP + TN + FP + FN}},$$

12 $$TPR = \frac{TP}{{TP + FN}},$$

13 $$TNR = \frac{TN}{{TN + FP}}.$$

## Results and discussion

Here, two experiments are executed to specify the best procedure for auditory attention detection using dynamic state analysis of the brain. First, the statistical analysis is performed on all single and multi-variate features to find the significant features ($p\_value < 0.05$) between the two groups. Then, classical and modern approaches mentioned in section "Literature Review" are utilized to assess the best proficiency for each single and multivariate feature set. Finally, the impact of the several durations of EEG segments is assayed on the performance of the proposed AAD method.

### Statistical analysis

As a preliminary data analysis, Kolmogorov–Smirnov (KS) test^89^ is used to measure the normality of feature vectors. Here, the probability values ($p < 0.05$) indicate that the data has non-normal distributions. Therefore, the Mann–Whitney U (Wilcoxon rank sum) test is selected to compare differences in extracted features between two independent groups when data is not distributed in normal form. KS test as s non-parametric test allows us to check whether the statistics at hand take different values from two different populations. $p\_value < 0.05$ indicates higher significance in terms of large differences in medians of the two groups. Tables 3 and Table 4 depict the significant *p*_values for every single microstate and RP features extracted from preprocessed EEG signals during the selective auditory attention task, respectively. According to these results, directly extracted features from EEG signals are not significant between the two cognitive tasks except *V*_max_. However, Table 5 shows the *p*_values of the multivariate features. Here, features including RR, DET, *V*_max_, and RPDE which are extracted from occurrence, duration, coverage, and mean GFP show significant differences between two auditory attention tasks, attending to ‘Spk 1’ vs attending to ‘Spk 2’.

**Table 3:** *P*-values of Mann–Whitney test for extracted microstate (MS) features directly from preprocessed EEGs. The symbol ^*^ indicates a significant difference ($p - value < 0.05$).

		Occurrence	Duration	Coverage	Mean GFP
DTU	Attending to ‘Spk1’ vs. Attending to ‘Spk2’	0.056	0.147	0.892	0.223
KUL	Attending to ‘Spk1’ vs. Attending to ‘Spk2’	0.004*	0.054	0.084	0.078

Significant values are in [bold].

**Table 4:** *P*-values of Mann–Whitney test for extracted features of recurrence plot (RP) directly from EEGs. The symbol ^*^ indicates a significant difference ($p - value < 0.05$).

		RR	DET	*L*_MEAN_	*L*_MAX_	ENTR	TT	*V*_max_	RPDE
DTU	Attending to ‘Spk1’ vs. Attending to ‘Spk2’	0.065	0.084	0.205	0.311	0.433	0.540	0.018*	0.220
KUL	Attending to ‘Spk1’ vs. Attending to ‘Spk2’	0.015*	0.043*	0.169	0.403	0.189	0.240	0.015*	0.431

Significant values are in [bold].

**Table 5:** *P*-values of Mann–Whitney test for multivariate features “MS + RP” extracted from EEG (note: eight RQA features are extracted from four microstates). The symbol ^*^ indicates a significant difference ($p - value < 0.05$).

		Occurrence	Duration	Coverage	Mean GFP
DTU	RR	0.011*	0.035*	0.030*	0.001*
	DET	0.032*	0.019*	0.128	0.154
	L_MEAN_	0.491	0.275	0.643	0.917
	*L*_MAX_	0.241	0.356	0.698	0.151
	ENTR	0.271	0.695	0.501	0.620
	TT	0.382	0.192	0.685	0.522
	*V*_max_	0.006*	0.040*	0.05	0.009*
	RPDE	0.026*	0.050	0.074	0.008*
KUL	RR	0.012*	0.119	0.037*	0.005*
	DET	0.094	0.120	0.189	0.019*
	L_MEAN_	0.636	0.071	0.190	0.207
	*L*_MAX_	0.068	0.106	0.520	0.198
	ENTR	0.042*	0.422	0.479	0.644
	TT	0.081	0.518	0.301	0.256
	*V*_max_	0.017*	0.205	0.049	0.081
	RPDE	0.001*	0.029*	0.123	0.005*

Significant values are in [bold].

### AAD

**Figure 6:**
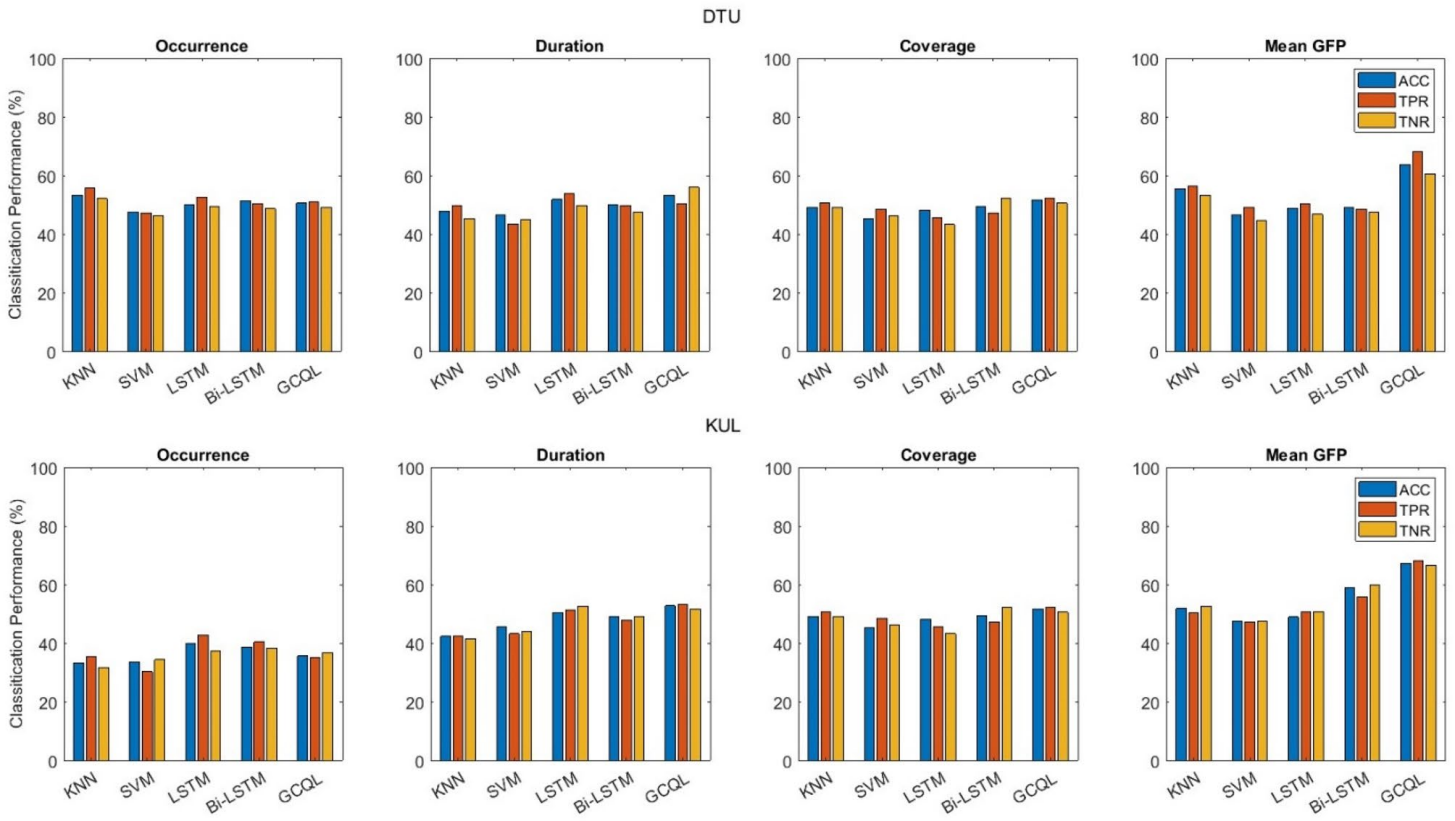
AAD performance only with MS feature vectors on DTU and KUL databases.

**Figure 7:**
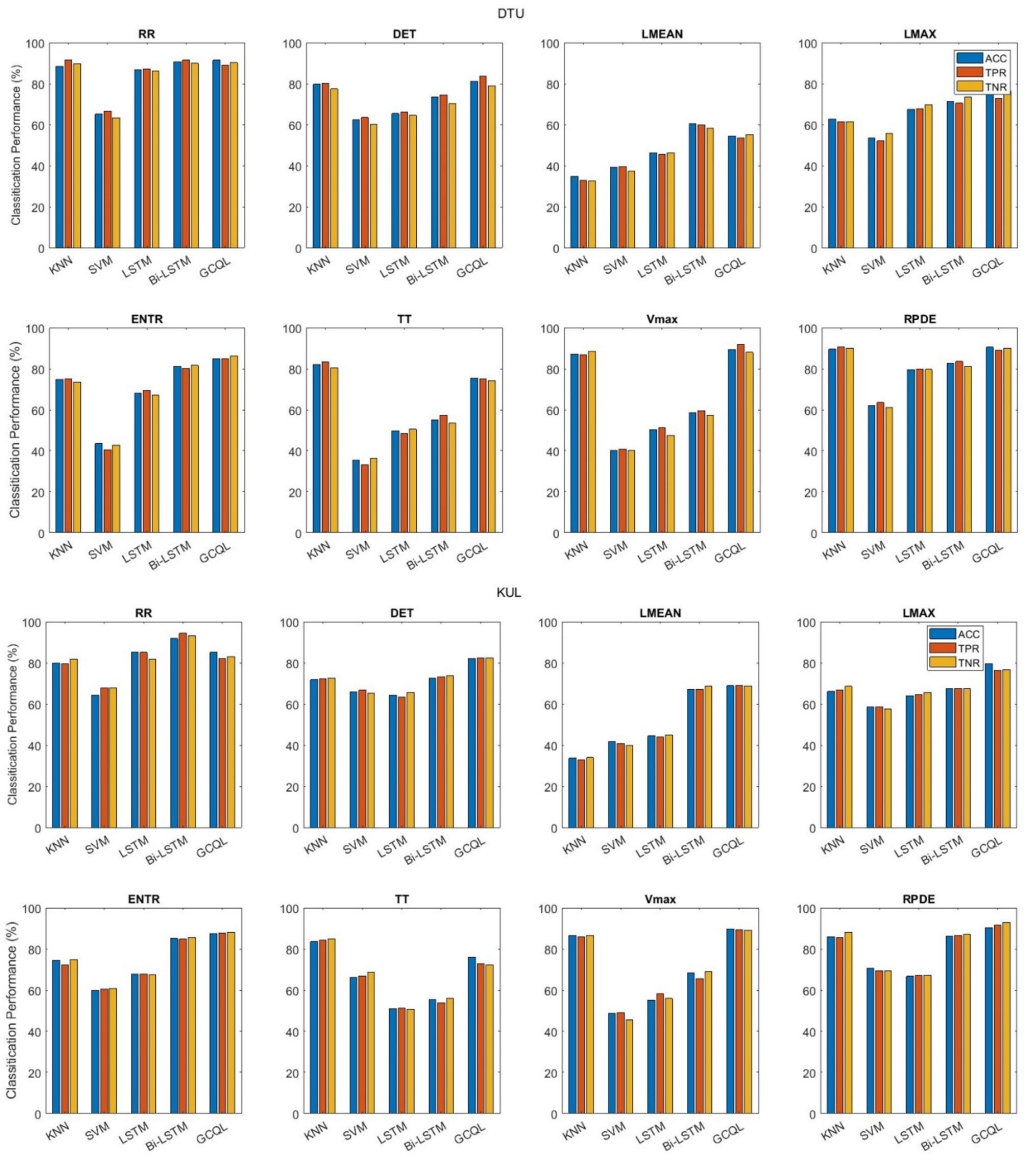
AAD performance only with RP feature vectors among all subjects on DTU and KUL databases.

Classification results acquired from microstate analysis are presented in Fig. 6 with occurrence, duration, coverage, and mean GFP parameters. These results include the ACC, TPR, and TNR on EEG signals divided into 1 s segments. It can be seen that these parameters fail in classification performance among two groups of attending to ‘Spk1’ and attending to ‘Spk2’ with accuracy close to the chance level. However, the highest accuracy is achieved by the “Mean GFP feature + GCQL classifier” compared with the other microstate parameters and classifiers. Figure 7 illustrates the attention detection for classifying with only RP parameters extracted from each 1 s segment of EEG signals. According to the figure, the highest ACC is 91.5% achieved by the “RR feature + GCQL classifier”.

In order to obtain the highest performance of AAD, the best features of the microstates and RP analysis are selected from the point of view of classification accuracy. Therefore, the recurrence rate of the mean GFP feature is calculated as an optimal multivariate feature and fed to the GCQL classifier.

**Figure 8:**
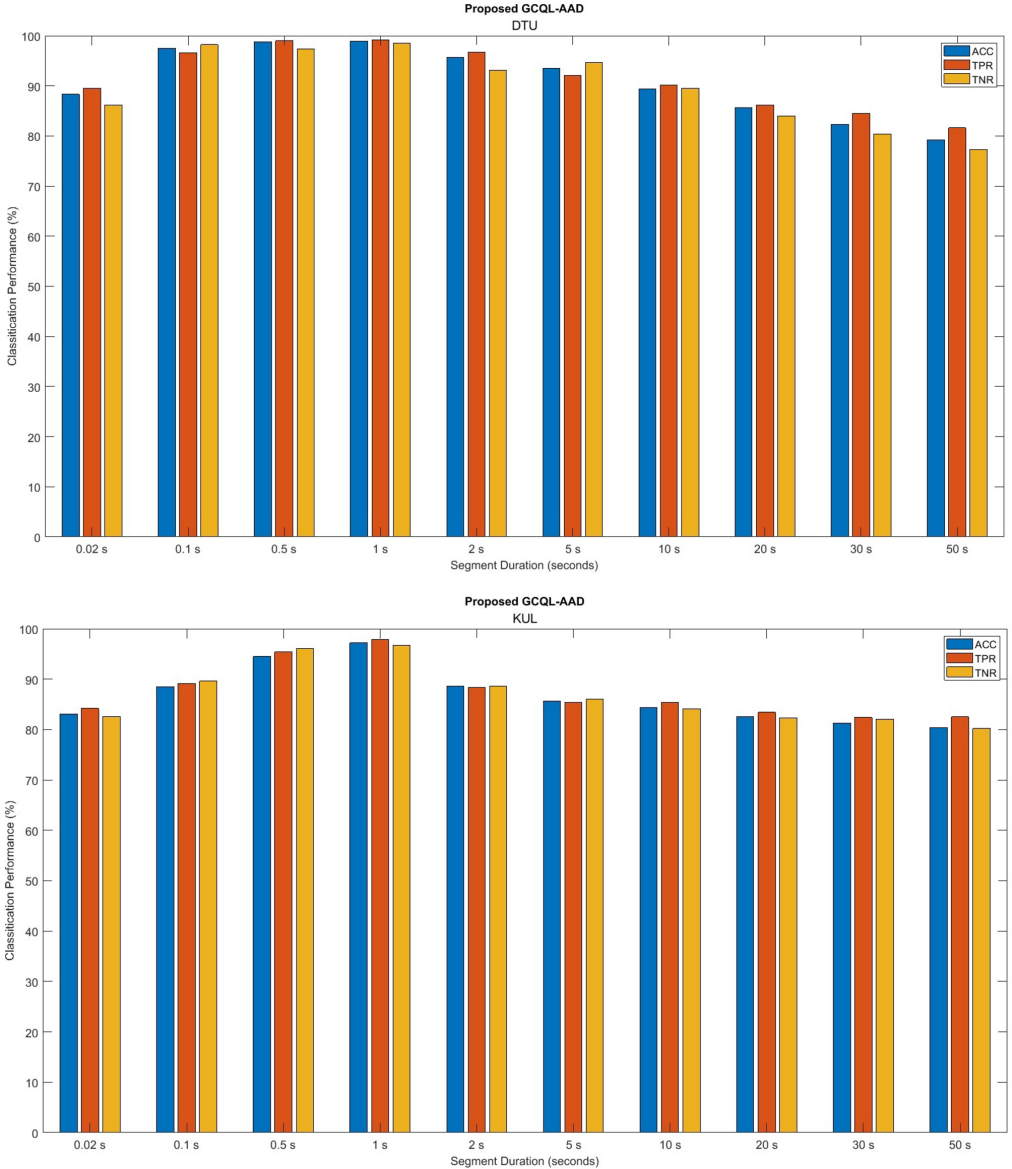
Performance of the proposed GCQL-AAD using multivariate feature (i.e., “mean GFP + RR”) and GCQL classifier for different EEG segments on DTU and KUL databases.

In the further experiment, the performance of the proposed method is examined for different segments of EEGs. To this aim, first, the mean GFP of EEG data is calculated by microstate analysis for different durations of EEG segments from 0.02 s to 50 s. Then, the recurrence rate (RR) is extracted from the mean GFP and given to the GCQL classifier. The average of the proposed AAD performances is shown in Fig. 8 for 100 epochs. It can be observed that the detection performance of the proposed algorithm decreases significantly as the duration of the EEG data increases. In addition, this figure illustrates that the measures of ACC, TPR, and TNR are increased as the data length is shortened, specifically, in the length of 0.1 s to 1 s. Moreover, the TPR and TNR values lie in acceptable ranges for all EEG segments. This achievement could be considered in online applications such as neuro-steered hearing aid devices.

**Figure 9:**
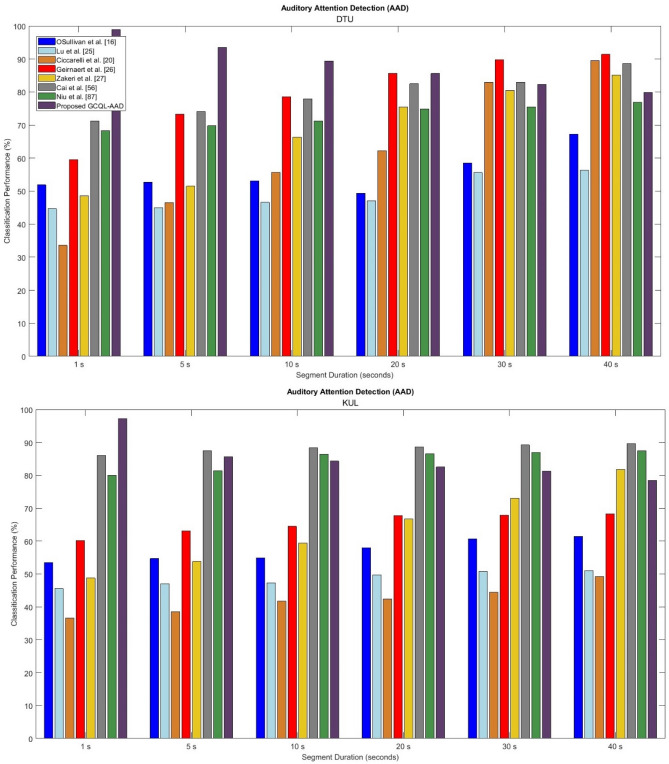
Comparison of the proposed AAD method (purple) with the others including “O’Sullivan et al^16^. (blue), Lu et al^25^. (light blue), Ciccarelli et al^20^. (orange), Geirnaert et al^26^. (red), Zakeri et al^27^. (yellow), Cai et al^56^. (neutral), and Niu et al^87^. (green) in terms of accuracy.

Figure 9 compares the performances of the proposed AAD method based on the optimal feature set (i.e., mean GFP + RR”) and GCQL classifier beside the baseline systems in terms of ACC measures. According to the accuracy criteria, the introduced AAD algorithm has superior proficiency than the baseline systems including “O’Sullivan et al^16^., Lu et al. ^25^., Ciccarelli et al^20^., Geirnaert et al^26^., Zakeri et al^27^., Cai et al^56^., and Niu et al^87^., It is observed that the accuracy of the baseline systems is increased with the increasing length of EEG durations, in general. In Table 6, the highest performances belong to the DTU database analysis for AAD. Here, the proposed system introduced by “O’Sullivan et al^16^. achieved accuracies of 51.9%, 52.7%, 53.1%, 49.4%, 58.5%, and 67.3% for window lengths 1 s, 5 s, 10 s, 20 s, 30 s, and 40 s, respectively. The proposed system introduced by Lu et al. ^25^. achieved accuracies of 44.7%, 45.0%, 46.6%, 47.1%, 55.6%, and 56.3% for window lengths 1 s, 5 s, 10 s, 20 s, 30 s, and 40 s, respectively. The AAD model proposed by Ciccarelli et al^20^. obtained accuracies of 33.6%, 46.5%, 55.7%, 62.2%, 83.0%, and 89.5% for EEG signals with window lengths 1 s, 5 s, 10 s, 20 s, 30 s, and 40 s, respectively. The AAD model introduced by Geirnaert et al^26^., achieved accuracies of 59.5%, 73.3%, 78.6%, 85.7%, 89.8%, and 91.4% for EEG signals with window lengths 1 s, 5 s, 10 s, 20 s, 30 s, and 40 s, respectively. The Zakeri et al^27^., AAD model obtained accuracies of 48.6%, 51.5%, 66.4%, 75.5%, 80.5%, and 85.2% for EEG signals with window lengths 1 s, 5 s, 10 s, 20 s, 30 s, and 40 s, respectively. The AAD model proposed by Cai et al^56^., obtained accuracies of 71.2%, 74.1%, 77.9%, 82.5%, 83.0%, and 88.6% for EEG signals with window lengths 1 s, 5 s, 10 s, 20 s, 30 s, and 40 s, respectively. The Niu et al^87^., AAD model achieved accuracies of 68.3%, 69.8%, 71.2%, 74.9%, 75.5%, and 76.9% for EEG signals with window lengths 1 s, 5 s, 10 s, 20 s, 30 s, and 40 s, respectively. However, the proposed method achieved ACC 98.9%, 93.5%, 89.4%, 85.6%, 82.3%, and 79.9% for EEG length 1 s, 5 s, 10 s, 20 s, 30 s, and 40 s where the highest ACC is for the short length of EEG signals on DTU database (i.e., 1 s segment).

**Table 6:** Comparison of the proposed AAD method with others including “O’Sullivan et al^16^., Lu et al. ^25^., Ciccarelli et al. ^20^., Geirnaert et al^26^., Zakeri et al^27^., Cai et al^56^., and Niu et al^87^. in terms of accuracy (%) for different length of EEG signals.

		1 s	5 s	10 s	20 s	30 s	40 s
DTU	O’Sullivan et al. ^16^	51.9	52.7	53.1	49.4	58.5	67.3
	Lu et al. ^25^	44.7	45.0	46.6	47.1	55.6	56.3
	Ciccarelli et al. ^20^	33.6	46.5	55.7	62.2	83.0	89.5
	Geirnaert et al. ^26^	59.5	73.3	78.6	85.7	89.8	91.4
	Zakeri et al. ^27^	48.6	51.5	66.4	75.5	80.5	85.2
	S. Cai et al. ^56^	71.2	74.1	77.9	82.5	83.0	88.6
	Y. Niu et al. ^87^	68.3	69.8	71.2	74.9	75.5	76.9
	Proposed GCQL-AAD	98.9	93.5	89.4	85.6	82.3	79.9
KUL	O’Sullivan et al. ^16^	53.5	54.7	54.9	58.0	60.7	61.4
	Lu et al. ^25^	45.6	47.1	47.3	49.8	50.8	51.0
	Ciccarelli et al. ^20^	36.6	38.6	41.8	42.4	44.5	49.2
	Geirnaert et al. ^26^	60.2	63.1	64.5	67.8	67.9	68.3
	Zakeri et al. ^27^	48.8	53.8	59.4	66.7	73.1	81.8
	S. Cai et al. ^56^	86.1	87.5	88.4	88.6	89.2	89.7
	Y. Niu et al. ^87^	80.0	81.4	86.4	86.6	87.0	87.5
	Proposed GCQL-AAD	97.2	85.7	84.3	82.5	81.3	78.5

Although the exploratory analysis yielded significant results for the short length of EEG signals, 1 s, it was the one where differences in microstates analysis had a stronger impact on extracting differences of brain function during the milliseconds. In addition, extracting RQA from microstates as multivariate features emphasizes the dynamic behavior of the brain performance throughout the auditory attention task. As well as the ability of the GCQL-AAD model to categorize auditory attention and extract temporal features and real-time analysis is one of the other advantages of the proposed model that has not been included in previous studies.

However, the results of the present study should be viewed in light of some limitations. First, the study was not designed for multi-talker scenarios with more than two talkers, so the proposed model could be confounded by the other attended speaker in the presence of two or more talkers. Second, this algorithm has been performed by high-dense scalp EEG which is not portable for real application. Using the smaller number of electrodes which have more relationship with the auditory attention cortex could enhance the ability of the proposed method to utilize in BCI devices.

## Conclusion

In the present work, a novel approach for auditory attention detection is presented based on the microstates and recurrence quantification analysis from EEG signals. Here, participants listen to the two talkers and focus on only one of them (during the half of trials, they attend to speaker number 1 and the rest attend to speaker number 2). In the first step, microstate analysis is performed to extract appropriate features from EEG states. Also, recurrence quantification analysis is utilized on the EEG signals to the emerging complex behavior of the brain. Then, the extracted features are given to the five types of classifiers (i.e., KNN, SVM, LSTM, Bi-LSTM, and GCQL) both individually and in combination to find the optimized AAD structure. Results of the experiments show that the extracting recurrence ratio (RR) from the mean of the global field power (mean GFB) with the GCQL classifier yields a higher performance, 98.9% in terms of accuracy.

The proposed AAD model has an important advantage over the forward and backward mapping algorithms, in the sense that attention recognition is performed from the EEG data of each listener without any access to auditory stimuli. Furthermore, the classification proficiency indicates that the proposed GCQL-AAD method performs higher than the recently published AAD approaches of O’Sullivan et al^16^., Lu et al^25^., Ciccarelli et al^20^., Geirnaert et al^26^., Zakeri et al^27^., Cai et al^56^., and Niu et al^87^., as the bassline systems. Additionally, the decision time window of EEG-based auditory attention detection is generally more than 1 s for previous research. Obtaining the best decoding performance in a shorter time window is an urgent application requirement. The present work could capture AAD with high performance in a shorter window length of EEG. In^20,26^, methods based on deep learning, especially CNN, have dominated the field of EEG decoding in AAD. However, using only CNN has limitations in high global dependence on capturing long-term sequences and detecting auditory attention through dynamic EEG signals. Therefore, we proposed an AAD-GCQL model by capturing the dynamic behavior of the brain to address the problem of temporal and dynamic dependencies. The experimental results confirmed the effectiveness of the proposed AAD architecture, which outperformed the other baseline models.

In this research, EEG signals of all recording electrodes (i.e., 64 channels) are used in the AAD analysis. To alleviate the computational load and time cost of the AAD algorithm, the number of EEG recording channels could be reduced by electrode reduction methods. The current work uses an experimental configuration only with two competing talkers which limits the applicability of the proposed algorithm. It is necessary to examine AAD efficiency for more realistic scenarios such as the cocktail party with many speakers.

## Data Availability

Any data that support the findings of this study are included within the article.
